# Update on Infections in Primary Antibody Deficiencies

**DOI:** 10.3389/fimmu.2021.634181

**Published:** 2021-02-11

**Authors:** Yesim Yilmaz Demirdag, Sudhir Gupta

**Affiliations:** Division of Basic and Clinical Immunology, Department of Medicine, University of California, Irvine, Irvine, CA, United States

**Keywords:** primary immunodeficiencies, infections, common variable immunodeficiency, immunoglobulin therapy, selective IgA deficiency, specific antibody deficiency, IgG subclass deficiency, selective IgM deficiency

## Abstract

Bacterial respiratory tract infections are the hallmark of primary antibody deficiencies (PADs). Because they are also among the most common infections in healthy individuals, PADs are usually overlooked in these patients. Careful evaluation of the history, including frequency, chronicity, and presence of other infections, would help suspect PADs. This review will focus on infections in relatively common PADs, discussing diagnostic challenges, and some management strategies to prevent infections.

## Introduction

Primary immunodeficiencies (PIDs), also known as inborn errors of immunity (IEI), are genetic disorders classically characterized by increased susceptibility to infections. According to the recent International Union of Immunologic Societies (IUIS) report, primary antibody deficiencies coalesce under the 3^rd^ category: Predominantly antibody deficiencies (PADs) (**Table 1**) (1). These immune deficiencies occur due to defects in B cell development or function. They are the most common types of IEIs worldwide (2–4). Patients have low levels of one or more immunoglobulin isotypes and/or inadequate production of pathogen-specific antibodies, and they develop infectious and noninfectious manifestations (**Table 2**) (5, 6, 23, 37, 45).

**Table 1:** Classification of inborn errors of immunity according to the 2019 IUIS report.

IUIS Classification Group	Primary Immunodeficiency Disease Category
I	Immunodeficiencies affecting cellular and humoral immunity
II	Combined immunodeficiences with associated or syndromic features
III	Predominantly antibody deficiencies
IV	Diseases of immune dysregulation
V	Congenital defects of phagocyte number of function
VI	Defects in intrinsic or innate immunity
VII	Autoinflammatory disorders
VIII	Complement deficiencies
IX	Bone marrow failure
X	Phenocopies of inborn errors of immunity

**Table 2:** Common and rarely reported infections in predominantly antibody deficiencies.

Disease (inheritance)	Gene	Bacterial infections	Viral infections	Fungal/protozoal infections	Other infections reported rarely*
**Severe reduction in all serum immunoglobulin isotypes with profoundly decreased or absent B cells, agammaglobulinemia**					
**X-linked agammaglobulinemia (XLA)**					
Bruton agammaglobulinemia	*BTK*	Respiratory tract infections with *H. influenza, S. pneumonia*, *Pseudomonas* spp, *Staphylococcus spp*, *H. parahemolytica*, *H. parainfluenza, Klebsiella* spp (5–8). Gastrointestinal infections with *Campylobacter*, *Salmonella* spp*, C difficile*, *H.pylori, Shigella* spp, *Flexispira (* 5–8 *).* Meningitis/sepsis with *S. pneumonia*, *H. influenza type b* (5, 6) Skin infections by *S. aureus* (6) Septic arthritis by *Mycoplasma*, *Ureaplasma* *(* 9)	Gastrointestinal infections with *Rotavirus*, Enterovirus (5) Vaccine-associated polio, encephalitis with *ECHO 11*, *Coxsackie* (5, 7) Hepatitis C (5, 6) Skin infections caused by Herpesvirus (6)	Gastrointestinal infections with *Giardia, Blastocistis hominis* (5, 6) Skin infections caused by *Candida sp* (6)	Disseminated infection by *Spiroplasma apis* (10), *Achromobacter xylosoxidans* sepsis (11), *Morganella morganii* pericarditis (12), Systemic infection by Aichi virus (13), Infective endocarditis by *Enterococcus faecalis* (14) Sepsis by *Pseudomonas* (7) Skin infections by non-*H. pylori* helicobacter (NHPH) (15)
**Autosomal recessive agammaglobulinemia**					
µ heavy chain deficiency	*IGHM*	Bacterial respiratory infections (16–18) *Pseudomonas* sepsis (16–18) Chronic diarrhea (18, 19)	Viral respiratory tract infections (17, 18) Enteroviral encephalitis (16) Vaccine-induced paralytic polio (18, 19)		*Haemophilus influenza arthritis* (20) Recurrent perirectal abscesses (16)
P110δ deficiency	*PIK3CD*	Recurrent sinopulmonary infections with encapsulated bacteria (21)			Persistent rotavirus enteritis (22) Catheter-associated *Klebsiella pneumonia* sepsis (22) *S. pneumonia* sepsis (22)
P85 deficiency	*PIK3R1*	Recurrent sinopulmonary infections with encapsulated bacteria (*H. influenza*, *S. pneumonia*) (23),	*Influenza A, CMV* (23) Prolonged diarrhea after Rotavirus vaccine		*Campylobacter spp* bacteremia, interstitial pneumonia (24)
SLC39A7 (ZIP7) deficiency	*SLC39A7*	Early onset infections (25)			
λ5 Deficiency	*IGLL1*				Otitis media at 2 months, meningitis caused by *Haemophilus* (26), bilateral lobar pneumonia at 3 months of age (27)
Igα deficiency	*CD79A*	Upper and lower respiratory tract infections, recurrent diarrhea (28)			HHV8 and JC virus encephalitis (29)
Igβ deficiency	*CD79B*	Upper and lower respiratory tract infections (30, 31)			Salmonella enteritis (30)
BLNK deficiency	*BLNK*	Upper and lower respiratory tract infections (32)			Enteroviral viremia, Pseudomonas sepsis, septic arthritis by *Proteus mirabilis* (32)
E47 transcription factor deficiency (AR or AD)	*TCF3*	Recurrent pneumonia and meningitis (33, 34)			Chronic diarrhea (34)
**Autosomal dominant agammaglobulinemia**					
Hoffman syndrome/TOP2B deficiency	*TOP2B*	Recurrent respiratory infections by encapsulated bacteria (35)			*Haemophilus influenza* meningitis (36)
**Severe reduction in At least 2 serum immunoglobulin isotypes with normal or low B cells, CVID phenotype**					
Common variable immune deficiency with no gene defect specified (CVID) (variable)	Unknown	Respiratory tract infections with *H. influenza, S. pneumonia*, mycoplasma, Moraxella, *Pseudomonas* spp, *Staphylococcus* spp, and *Klebsiella pneumonia* (37, 38) Gastrointestinal infections caused by *Campylobacter* sp, *Salmonella* sp, *Clostridium difficile*, *Escherichia coli* (37–39) Meningitis caused by *S. pneumonia*, *N. meningitides*, and *H. influenza* (37, 38) *Staphylococcal aureus* skin abscesses (37, 38)	Respiratory infections caused by human rhinovirus (*HRV*), A*denovirus*, respiratory syncytial virus (*RSV*), seasonal *Coronavirus* (40, 41) *Norovirus* (42), *CMV* (39) Recurrent *Herpes zoster*, invasive papillomavirus (37, 38)	Gastrointestinal infections caused by *Giardia lamblia* (37–39) *Blastocistis hominis*,	Mycobacterial disease, listeriosis, nocardia, Bacilli-Calmette-Guerin, *Molluscum contagiosum, Measles*, *HIV*, severe EBV, systemic adenovirus infection, Kaposi sarcoma, Hepatitis C, Hepatitis B, cerebral toxoplasmosis (37, 38) Gastrointestinal infections caused by *Isospora belli* (43), cryptosporidium, *Cryptococcus*, *Candida sp*, Histoplasmosis visceral mycosis (38)
Activated P110 δ syndrome (AD)	*PIK3CD* (GoF) *PIK3R1* (LoF)	Recurrent respiratory infections by encapsulated bacteria (21) Staphylococcal skin and dental abscess (21) *Clostridium difficile* colitis Recurrent conjunctivitis (44)	Recurrent sinopulmonary infections with viruses, *Influenza A* (44) Severe and persistent infections with EBV, CMV, HSV, VZV (44–46)	Chronic mucocutaneous candidiasis (44)	*Staphylococcus aureus*-related periorbital cellulitis, *Pseudomonas aeruginosa* septicemia, chronic *Giardia intestinalis* (44)
PTEN deficiency (AD)	*PTEN*	Recurrent upper respiratory infections (47)			Neonatal group B *Streptococcus* infection, *Pneumocystis jiroveci* pneumonia (48)
TWEAK deficiency (AD)	*TNFSF12*	Recurrent upper respiratory infections (49)	Warts (49)		Pneumococcal meningitis, Osteomyelitis (49)
TRNT1 deficiency (AR)	*TRNT1*	Upper and lower respiratory tract infections (50)			
NFKB1 deficiency (AD)	*NFKB1*	Upper respiratory tract infections with S. Pneumonia, H. Influenzae, *P. aeruginosa; Clostridium difficile* colitis (51, 52) Bacterial meningitis (52)	Shingles; vaginal HPV; invasive CMV; EBV (51, 52)	*P. jiroveci* pneumonia; *Mycobacterium avium intracellulare; Giardia* (51, 52)	PML (51); Chronic Norovirus, rhinovirus (52)
NFKB2 deficiency (AD)	*NFKB2*	Upper respiratory tract infections (53)	Recurrent HSV skin infections, severe VZV, molluscum, EBV, CMV infections (53)	*Pneumocystis jiroveci*, *Giardia*, oral candidiasis (53)	Toxoplasmosis; onycomycosis; *Salmonella* (53)
IKAROS deficiency (AD)	*IKZF1*	Recurrent sinopulmonary infections starting in infancy (54) Sepsis, pneumonia, skin abscess by *P. aeruginosa* (55)	Severe RSV bronchiolitis, Adenoviral pneumonia, recurrent HSV, EBV+, HPV genital infections (54, 55)	*Pneumocystis jiroveci* pneumonia, recurrent oral candidiasis (55)	Lung infection with *Klebsiella sp*, Aspergillus sp, Mycobacterium avium complex. Pneumococcal meningitis, Cryptosporidial cholangitis, *Candida parapsilosis* fungemia (55), *Enterococcus gallinarum* sepsis (56)
ATP6AP1 deficiency (XL)	*ATP6AP1*	Recurrent respiratory infections starting in infancy (57)			
Mannosyl-oligosaccharide glucosidase deficiency (AR)	*MOGS*	Bacterial respiratory infections (58)			Urinary tract infection by Escherichia coli, Pneumococcal pneumonia with empyema*; Staphylococcus aureus* osteomyelitis (58)
CD19 deficiency (AR)	*CD19*	Recurrent upper and lower respiratory tract infections (59)			Pneumococcal meningitis; Chronic gastritis by *Heliobacter pylori* (59)
CD81 deficiency (AR)	*CD81*	Recurrent respiratory tract infections (60) (1 of 1 reported patient)			
CD20 deficiency (AR)	*CD20*	Recurrent upper and lower respiratory tract infections (61) (1 of 1 reported patient)			
CD21 deficiency (AR)	*CD21*	Recurrent respiratory tract infections with encapsulated bacteria (62)			
TACI deficiency** (AR or AD)	*TNFRSF13B*	Recurrent respiratory tract infections (63, 64)	Recurrent viral respiratory infections (64)		Rectal herpes simplex (64)
BAFF receptor deficiency (AR)	*TNFRSF13C*	Recurrent upper and lower respiratory tract infections (65)			Severe *Herpes zoster* (65)
IRF2BP2 deficiency (AD)	*IRF2BP2*	Recurrent upper and lower respiratory tract infections (66)			
ARHGEF1 deficiency (AR)	*ARHGEF1*	Recurrent upper and lower respiratory tract infections (67)			Recurrent VZV, severe oral HSV (67)
SH3KBP1 deficiency (XL)	*SH3KBP1*	Recurrent upper and lower respiratory tract infections (68)			
SEC61A1 deficiency (AD)	*SEC61A1*	Early-onset, severe, bacterial sinopulmonary infections; gastroenteritis (69)			
RAC2 deficiency (AR)	*RAC2*	Recurrent sinopulmonary infections (70)			
**Severe reduction in serum IgG and IgA with normal/elevated IgM and normal numbers of B cells, hyperIgM phenotype**					
AID deficiency (AR or AD)	*AID*	Upper and lower respiratory tract infections, skin infections, meningitis (71)		Giardia infections (71)	HSV encephalitis, Hepatitis B (71)
UNG deficiency (AR)	*UNG*	Upper and lower respiratory tract infections (72)			
MSH6 deficiency (AR)	*MSH6*	No recurrent or severe infections reported to date (73)			
INO80 deficiency (AR)	*INO80*	Severe and recurrent bacterial respiratory infections (74)			
**Isotype, light chain, or functional deficiencies with generally normal numbers of B cells**					
Ig heavy chain mutations and deletions (AR)**	Mutation or deletions at 14q32	Upper and lower respiratory tract infections	Enteroviral infections		
Kappa chain deficiency (AR)**	*IGKC*	Upper and lower respiratory tract infections (75)			
Isolated IgG subclass deficiency and IgG subclass deficiency with IgA deficiency ** (IgGSCD)	Unknown	Upper and lower respiratory tract infections with *S. pneumoniae*, *H. influenzae type b*, *N. meningitides*	Upper and lower respiratory tract infections with viruses		
Selective IgA deficiency** (SIAD)	Unknown	Upper and lower respiratory tract infections with *S. pneumoniae*, *H. influenzae;* Pharyngitis; urinary tract infections (76, 77)	Upper and lower respiratory tract infections with viruses Stomatitis, herpes labialis (76, 77)	*G. lamblia* gastroenteritis (78)	
Specific antibody deficiency ** (SPAD)	Unknown	Bacterial upper and lower respiratory tract infections (79) Chronnic otorrhea in children (79, 80)			Recurrent *HSV*, *S. pneumonia* sepsis, recurrent folliculitis (79)
Transient hypogammaglobulinemia of infancy ** (THI)	Unknown	Upper and lower respiratory tract infections, abscesses, urinary tract infections, meningitis, sepsis (81)			Hepatitis, osteomyelitis, *VZV* infection (81)
CARD11 GoF (AD) **	*CARD11*	Recurrent upper and lower respiratory tract infections, cellulitis (82)	*M. contagiosum*, Chronic *EBV*, *BK virus* (82)		*S. pneumoniae* bacteremia (82)
Selective IgM deficiency ** (SIGMD)	Unknown	Upper and lower respiratory tract infections with *S. pneumoniae*, *H. influenza* and viruses (83–85)	*Varicella zoster*, CMV, aseptic meningitis (85)	*G. lamblia*, Mycobacterial infection (85)	Sepsis (*S. pneumonia*, *N. Meningitides*) aseptic meningitis, Invasive *Aspergillus* (85, 86)

AR, autosomal recessive; AD, autosomal dominant; CDG-IIb, congenital glycosylation disorder type IIb; CMV, Cytomegalovirus; EBV, Ebstein-Barr virus; GoF, gain-of-function; LoF, loss-of-function; XL, X-linked.

*Infections reported < 10 cases in relatively common PADs, such as CVID, SIAD, IgGSCD, SPAD, and XLA, and <2 patients in all other PADs.

**These conditions may be asymptomatic.

Early diagnosis and timely initiation of immunoglobulin replacement therapy (IgRT) may prevent infections, and therefore, alter the clinical course of PADs. Although sinopulmonary infections with encapsulated bacteria are the most common infections, PADs may also present with infections caused by other usual or unusual microorganisms affecting various organs. A thorough understanding of the types of infections that these patients are susceptible will be invaluable to suspect PADs and for early diagnosis. Here, we will summarize best known and relatively common PADs while focusing on their infectious manifestations. We will use primary antibody deficiencies and predominantly antibody deficiencies interchangeably.

Specific PADs are grouped into four subcategories based on their immunologic findings.

### Severe Reduction in All Serum Immunoglobulin Isotypes With Profoundly Decreased or Absent B Cells: Agammaglobulinemia

These conditions are associated with circulating B cells below 2% of total lymphocytes, and undetectable or very low levels of all isotypes of serum immunoglobulins. About 85%–90% of patients in this group have X-linked agammaglobulinemia (XLA) due to mutations in the Bruton tyrosine kinase gene (BTK) (87), and 5% of patients have immunoglobulin μ heavy chain deficiency.

#### X-Linked Agammaglobulinemia

Classically, affected boys begin to suffer from infections at 4 to 6 months of age. Late-onset and milder cases have also been reported (88, 89). The estimated incidence ranges from 1:200,000 to 1:100,000 live births (90). Neutropenia is seen in 11% to 22% of patients, which resolves once the appropriate IgRT is initiated (5). Normal IgA and IgM levels have been reported in a few patients (5, 7).

Sinopulmonary infections are the most common infections in XLA, but gastrointestinal infections, and more invasive infections may also occur (**Table 2**). For exampleanalyses of large cohorts revealed that up to 8% of patients may present with meningitis caused by *Streptococcus pneumoniae*, *Neisseria meningitides*, and *Haemophilus influenzae*, or encephalitis caused by *ECHO* virus, *Coxackie* virus, and poliovirus (5–8). Enteroviruses also cause dermatomyositis-like presentation (5, 91). Enteroviral infections have been particularly challenging for patients with XLA because they are usually chronic, systemic, resistant to therapy, and may be fatal.

In a US registry consisted of 201 patients with XLA, *Giardia lamblia* was the most common etiology of gastrointestinal infections, and *Pseudomonas spp* was the most common etiology of sepsis, accounting for 12 of 46 and 6 of 29 patients, respectively (5).

Recently, refractory cellulitis, pyoderma gangrenosum, and bacteremia due to non-*Helicobacter pylori Helicobacter* (NHPH) such as *Helicobacter bilis (Flexispira*) and *Helicobacter cinaedi* have been reported in XLA (15, 92). In addition, case reports describing septic arthritis due to *Mycoplasma* or *Ureoplasma* (9), a disseminated infection caused by *Spiroplasma apis* (a honey bee pathogen) (10), sepsis due to *Achromobacter xylosoxidans* mimicking juvenile idiopathic arthritis (11), *Morganella morganii* pericarditis (12), and infective endocarditis due to *Enterococcus faecalis* (14) have been published. Aichi virus, a common contaminant in ponds, sewages, and shellfish, causing self-limiting gastroenteritis in immunocompetent persons, may cause chronic and severe infection including fever, bloody diarrhea, chronic hepatitis, and splenomegaly in XLA (13).

#### Autosomal Recessive and Autosomal Dominant Agammaglobulinemia

Immunoglobulin μ heavy chain (IGHM) deficiency is associated with more severe clinical manifestation compared to XLA (16, 17, 19). Like in XLA, neutropenia may be seen in 30% of these patients (16, 19). In addition to bacterial respiratory tract infections, these patients are at increased risk for pseudomonas sepsis, arthritis, skin abscesses, chronic diarrhea, and enteroviral central nervous system (CNS) infections (16–19). In a study on 19 patients with IGHM, 7 patients had significant enteroviral infections, and 4 had pseudomonas sepsis before the IgRT was started (19).

Other PADs in this group are extremely rare and include defects in phosphatidylinositol 3–kinase δ (PI3Kδ) signaling pathway, which include *PIK3CD* and *PIK3R1* deficiencies, encoding catalytic and regulatory subunits of PI3Kδ, respectively. Biallelic loss of function (LoF) mutations in these genes cause early-onset bacterial sinopulmonary infections, viral infections, oral thrush, esophageal candida infection, *Campylobacter* bacteremia as well as transient neutropenia and thrombocytopenia (**Table 2**) (21, 23, 24, 93).

### Severe Reduction in at Least 2 Serum Immunoglobulin Isotypes With Normal or Low B Cells: CVID Phenotype

#### Common Variable Immunodeficiency (CVID)

As the most common symptomatic IEI, CVID affects 1:50,000 to 1:10,000 people (4, 37, 94, 95). Patients are usually diagnosed between the 3^rd^ and the 5^th^ decades. According to the European Society for Immunodeficiencies Database (ESID), 33.7% of patients had the onset before 10 years, and, in a large US cohort, 28% of patients had the diagnosis before 21 years (37, 94). The most common clinical manifestations include infections and infection-related complications such as bronchiectasis (37, 38, 94). Otitis media, failure to thrive, and developmental delay were reported more in pediatric-onset CVID compared to adult-onset (96). Non-infectious manifestations, such as autoimmunity, may precede infections and, they are associated with increased mortality (38).

Because of the significant clinical and immunological heterogeneity, the definition of CVID has undergone several revisions. According to the most recent consensus, the diagnosis should be based on laboratory findings, which include low IgG (< 2SD for age-adjusted levels), low IgA, and/or low IgM, and suboptimal response to T-cell-independent or T-cell-dependent vaccines. Some patients may also have low CD4^+^ T cells. Other conditions such as medications, protein loss, and various disorders that may cause low immunoglobulins should be excluded (97).

The most common causes of pneumonia in CVID are S*. pneumonia* and *H. influenza*, followed by *Mycoplasma pneumonia*, *Pseudomonas spp*, *Staphylococcus spp*, and *Klebsiella pneumonia* (37, 38) (**Table 2**). The frequency and severity of respiratory infections decrease with IgRT. However, despite IgRT, a prospective study showed that respiratory infections may still occur more commonly in patients with PADs and, common circulating upper respiratory viruses may be detected more often in patients with PADs, including CVID, compared to healthy controls. The most common respiratory virus isolated in this study was human rhinovirus (HRV), followed by adenovirus, respiratory syncytial virus, and seasonal coronavirus (non-pandemic). Using a multiplex PCR approach, this study also showed that HRV and *H. influenzae* might frequently co-occur in patients with PADs (40). Furthermore, prolonged shedding of HRV after an acute infection has been shown by another group in patients with CVID and XLA compared to healthy controls (40 days versus 10 days) (41). *Pneumocystis jiroveci* pneumonia was reported in six patients with CVID, three were on systemic steroid treatment and, one was on systemic steroids and 6-mercaptopurine (37).

Large cohorts and observational studies reported gastrointestinal manifestations in 47% to 67% of patients with CVID. Gastrointestinal infections were seen up to 24% of patients with gastrointestinal symptoms (38, 39, 98). In a study on 50 patients with CVID and gastrointestinal symptoms, 14, six, and five patients developed infections by *G. lamblia*, *Clostridium jejuni*, and *Salmonella*, respectively (98). *G. lamblia* was also the most common gastrointestinal infection in other cohorts (38, 38). Undetectable serum IgA levels increase the risk of gastrointestinal infections (38). Although *Helicobacter pylori* is not a typical infection in CVID, perhaps due to recurrent antibiotics treatment, a recent study reported *H. pylori* infection in at least 26% of patients with gastrointestinal symptoms. Some of these patients had intestinal metaplasia, which persisted for at least 3 years after the treatment of *H. pylori* (98). Chronic diarrhea due to *Giardia lamblia* and *Isospora belli* triggered the CVID diagnosis in a 62-year-old woman (43).

Studies on large cohorts also revealed that *Norovirus* and *Cytomegalovirus* (CMV) were among the most common viral causes of gastrointestinal infections in CVID (38, 39, 98). Others reported that Norovirus can be very severe, causing malabsorption and enteropathy requiring parenteral nutrition (42, 99). Although most patients with PADs, efficiently clear oral polio vaccine (OPV)-related virus (100), viral shedding up to 28 years after OPV was reported in one patient (101).

Adenovirus, CMV, and EBV infections were the cause of death in eight of 13 patients in a cohort of 25 patients with CVID who received hematopoietic stem cell transplantation (HSCT) because of lymphoma or treatment-refractory immune dysregulation (102).

Recurrent shingles is the most common viral skin infections in CVID, and the risk factors include steroid therapy, chemotherapy, and older age (38, 103).

#### Activated Phosphoinositide 3-Kinase Delta Syndrome (APDS)

Activated phosphoinositide 3-kinase delta syndrome (APDS) is caused by autosomal dominant (AD) gain-of-function (GoF) mutations in *PIK3CD* (APDS1), AD loss-of-function (LoF) mutations in *PIK3R1* (APDS2) or *PTEN* (phosphatase and tensin homolog deleted on chromosome 10). More than 200 patients have been reported with APDS (21). Patients develop recurrent respiratory infections, usually caused by *S*. *pneumoniae* and *H*. *influenza* during childhood, followed by lymphoproliferation, progressive lymphopenia, autoinflammatory disease, early onset bronchiectasis, and lymphoma. Short stature and neurodevelopmental delay were reported in some cohorts (45, 104). According to these reports, about 20% of patients developed conjunctivitis, which progressed to preorbital cellulitis in some. Persistent Epstein-Barr virus (EBV) or CMV viremia, EBV lymphoproliferative disease, and CMV lymphadenitis are characteristics of APDS. Chronic EBV viremia was seen in all patients in a series of 9 with APDS (105). Other infections are summarized in **Table 2** (45, 104, 106, 107).

Disseminated CMV infection and chronic diarrhea due to vaccine-induced rotavirus were reported in one patient with APDS2 who had tolerated oral polio and Bacillus Calmette–Guérin (BCG) vaccines (23).

#### Other Monogenic Defects Associated With CVID Phenotype

Genetic defects may be responsible in up to 30% of patients with CVID who had at least one of the following criteria: younger age of onset, autoimmune/inflammatory conditions, low B cells, and/or family history of hypogammaglobulinemia (51). For example, mutations in the gene encoding TACI (transmembrane activator and calcium-modulator and cyclophilin ligand interactor) are among the most common genetic defects attributed to CVID phenotype. The inheritance pattern may be autosomal dominant or autosomal recessive with the characteristics including, like in many other primary immunodeficiencies, incomplete penetrance and variable expressivity. Mutations in *TACI* were found in 4 of 19 unrelated patients with CVID phenotype and one of 16 patients with selective IgA deficiency (SIAD) in one study (63). Another group reported *TACI* mutations in 26 of 176 patients with CVID phenotype (64). Interestingly, none of those patients had a family history of immune deficiency, and none of their 10 familial cases of CVID had *TACI* mutations. Clinically, patients with *TACI* mutations present with recurrent sinopulmonary infections as well as autoimmunity, and to date, studies suggest that clinical phenotypes of *TACI* mutations are influenced by additional genetic and environmental changes (108).

According to a recent large US CVID cohort, the second most common monogenic mutations following *TACI* mutations were autosomal dominant variants in nuclear factor κ B subunit 1 (*NFKB1*), accounting 14 of 235 patients (109). In addition to infections, the majority of these patients also had one or more of the following manifestations: lymphocytic/granulomatous infiltrate, enteropathy, and autoimmunity. In another cohort, infections in five patients with NFKB1 deficiency were summarized (51). In addition to sinopulmonary infections, these patients suffered from shingles, *P. jiroveci* pneumonia, *Clostridium difficile* colitis, *Mycobacterium avium intracellulare* (MAI), and progressive multifocal leukoencephalopathy (PML). The latter was the cause of death in 1 of 2 patients.

Heterozygous AD mutations in *IKZF1*, encoding the zinc-finger transcription factor IKAROS were recently reported in patients with CVID phenotype (54, 55, 110). These patients have progressive loss of serum immunoglobulins and B cells. While *S. pneumoniae* was a common pathogen, increased susceptibility to viral or fungal infections was not observed in these patients.

Other monogenic conditions associated with CVID phenotype have been reported in a limited number of patients. Although their clinical presentations are similar and consist of recurrent infections starting early in life, there are some deviations. For example, infections in BAFF receptor deficiency may start as late as 70 years of age (65). In addition, some defects are associated with specific clinical manifestations, such as hamartoma and macrocephaly in PTEN deficiency, sideroblastic anemia and progressive developmental delay in TRNT1 deficiency, and severe neurologic disease and dysmorphic facial features in mannosyl-oligosaccharide glucosidase deficiency (58, 111, 112).

### Severe Reduction in Serum IgG and IgA With Normal/Elevated IgM and Normal Numbers of B Cells: HyperIgM Phenotype

These conditions result from defects in class-switch recombination (CSR). Activation-induced deaminase (AID) plays a significant role in CSR and somatic hypermutation (SHM). Together, SHM and CSR are crucial for a pathogen-specific, high-affinity antibody response. Uracil-DNA glycosylase (UNG) also plays a significant role in CSR and SHM (113, 114). Consequently, defects in AID or UNG genes result in recurrent and severe infections, autoimmunity (AID deficiency), and lymphoma (UNG deficiency) (**Table 2**) (19, 71, 115).

In this group, MSH6 deficiency may not be associated with susceptibility to recurrent infections (73).

### Isotype, Light Chain, or Functional Deficiencies With Generally Normal Numbers of B Cells

#### Selective IgA Deficiency

Selective IgA deficiency (SIAD) is defined as undetectable serum IgA levels, and normal IgG and IgM levels after age 4 (116). The prevalence ranges from 1:18.500 in Eastern Asian population to 1:500 in Caucasians (116–118). Some chromosomal disorders, including chromosome 18 abnormalities, trisomy 21, and 22q11.2 microdeletion are associated with SIAD (119–123). In addition, familial clustering may occur (76, 124–126). SIAD may be transient in children, or sometimes, it may evolve to CVID (76, 127, 128).

Although, the majority of patients are asymptomatic, recurrent bacterial, and viral respiratory infections, autoimmunity (e.g., celiac disease), and allergies are seen more commonly in SIAD than in general population. In addition, individuals with SIAD more often undergo tonsillectomy and adenoidectomy, and they develop pharyngitis, stomatitis, herpes labialis, and urinary tract infections more often than the general population (76, 77, 129–131).

While the recommendation is to avoid a definite diagnosis of SIAD before age 4, one study showed that children with undetectable IgA levels suffered from infections more often than children with detectable but low IgA levels, and atopy was more common in both groups than in children with normal IgA levels (121).

In some patients, SIAD and specific antibody deficiency, or SIAD and IgG subclass deficiency may co-occur. Bronchiectasis and recurrent infections are more commonly reported in these patients than in patients with isolated SIAD (123).

Like in many other PADs, *G. lamblia* has been an increasingly reported gastrointestinal pathogen in SIAD (78, 131).

In patients older than 12 years, one study reported that, although the prevalence of *H. pylori*-associated dyspepsia was not higher in SIAD, those patients who had *H. pylori* infection experienced more esophagogastroduodenoscopy (EGD)-proven gastritis, duodenal ulcers, and nodular lymphoid hyperplasia (132). Other studies showed that *H. pylori* infection was one of the most common gastrointestinal infections in children (< 17 years) with SIAD (133, 134). In addition, *H. pylori* infection may be associated with more severe periodontitis in SIAD (129).

Interestingly, patients with SIAD are not susceptible to rotavirus disease, and in fact, they may develop higher levels of rotavirus-specific IgG1 than healthy individuals (135).

#### Specific Antibody Deficiency

Specific antibody deficiency (SPAD or SAD) is defined as an inadequate antibody response to polysaccharide antigens in patients who have normal serum immunoglobulin levels and IgG subclasses. It may be the most common PIDs in children (3), and it may be transient (79, 136). In addition to bacterial respiratory tract infections, very rarely, invasive infections and even bronchiectasis may develop in undiagnosed patients (**Table 2**) (136, 137).

#### IgG Subclass Deficiency

IgG subclass deficiency (IgGSCD) is diagnosed when one or more IgG subclass levels are 2SDs below the age-adjusted range in patients with normal total IgG levels. Patients present with recurrent bacterial and viral respiratory infections and atopy (3, 138–141). IgGSCD has also been reported more commonly in patients with chronic obstructive pulmonary disease (COPD) exacerbation (141).

In children, the most common IgGSCD is IgG2 deficiency followed by IgG3 deficiency, and maybe transient (142, 143). In adults, IgG3 deficiency may be the most common IgGSCD (144). IgG3 deficiency was also the most common immune deficiency in children and adults with refractory rhinosinusitis (145).

IgGSCD and SPAD may co-occur (140). In children, this combination may be more common in boys, may be associated with bronchiectasis, and may progress to CVID or other immunodeficiencies (143).

#### Selective IgM Deficiency

Selective IgM deficiency (SIGMD), defined as low serum IgM levels (< 2SD), and normal IgA and IgG levels, can be seen in children and adults (83, 146–148). Its prevalence was reported as 0.37% in healthy blood donors (147). About 45% of symptomatic patients may also have SPAD or IgGSCD (83, 84). Respiratory infections, and rarely, bronchiectasis, sepsis, and meningitis may be seen in up to 80% of symptomatic patients (**Table 2**) (84–86, 149).

In addition, atopic diseases and autoimmunity are common, especially in adults with SIGMD (85, 148).

## COVID-19 and PADs

Coronavirus disease -19 (COVID-19) caused by severe acute respiratory distress syndrome coronavirus 2 (SARS-CoV-2), has been one of the major pandemics in human history. Like other immunocompromised states, PADs are presumed to be a risk factor for severe SARS-CoV-2 infection. Though data are limited, a large study including PADs (N:53) who developed COVID-19 reported that 13 of 40 patients with PAD who also had associated co-morbidities died whereas all 13 patients who had no COVID-19 associated co-morbidities survived (150).

An effective and timely production of pathogen-specific neutralizing antibodies should prevent viral replication and progression to severe disease. Therefore, one would expect worse clinical outcome in patients with PADs. In addition, SARS-CoV-2-specific T cells were recently demonstrated to be important in controlling and resolution of COVID-19 (151). Recently, two young adults with XLA (one had underlying bronchiectasis) developed COVID-19 pneumonia, but did not require intensive care. Another three patients with XLA had prolonged COVID-19 with increased proinflammatory responses recovered after treatment with convalescent serum (152, 153). Another group reported that two patients with agammaglobulinemia due to absent B cells had milder infection than five patients with CVID who had detectable B cells (154). These observations may be explained by presence of normal T cells in the cases of XLA, and defective T cells in some patients with CVID, and emphasize the importance of T cells in COVID-19. Detailed examination of clinical and immunologic response to SARS-CoV-2 in patients with PADs will provide indispensable knowledge on the pathogenesis of COVID-19.

## Challenges in Diagnosing Infections in PADs

Although infections are the most common manifestations of PADs, identifying the pathogen is not always easy because of high chance of false-negative serologic testing due to defects in pathogen-specific antibody production. In addition, false-positive serology may be seen in patients who are on IgRT. Therefore, direct identification of the microorganism by blood or tissue cultures, immunofluorescense staining, pcr testing, or amplicon sequencing are necessary for accurate diagnosis. Recently, next-generation sequencing (NGS) and matrix-assisted laser desorption/ionization time-of-flight mass-spectrometry (MALDI-TOF MS) successfully identified the pathogens in patients with PADs (13, 15, 155).

## Relationship Between IgG Levels and Infections

Life-long IgRT, administered either intravenously (IVIG) or subcutaneously (SCIG), is the standard treatment of the majority of PADs (7, 139, 140). In general, IgRT does not prevent or treat non-infectious manifestations.

Serum IgG trough levels, defined as IgG levels just before the next dose, strongly correlate with the dose and interval (8, 156). However, some patients with bronchiectasis or chronic lung disease require twice as much IgRT dose to achieve the same IgG trough levels (103, 157). Bacterial infections and bronchiectasis are more commonly seen in patients with low IgG levels (64, 103, 158, 159). IgRT may not significantly reduce viral respiratory infections (40).

A meta-analysis showed a progressively reduced number of infections with increasing IgG trough levels from 660 to 960 mg/dL. Beyond this level, the reduction in infection rate was not statistically significant (156). In another study, a positive correlation between total IgG trough levels and specific anti- pneumococcus and anti- *H. influenza* IgG levels were observed, and no serious lung infections developed in patients with IgG trough levels ≥700 mg/dL (160). In XLA, IgG trough levels > 800 mg/dl may prevent the onset of bronchiectasis, chronic sinusitis, and enteroviral infections (8).

On the other hand, IgG trough levels that prevent bacterial infections varied from 5 g/L to 17 gr/L in different patients, suggesting that the dose should be individualized based on clinical symptoms (103, 161). However, adjusting the IgRT dose based on frequency of infections alone may not be sufficient to prevent chronic lung damage (8, 157, 162). In addition, very low serum IgA and IgM levels have been associated with more severe radiographic lung disease and higher chance of bacterial colonization (159, 163). These findings suggest that higher trough IgG levels should be aimed in selected patients, such as, patients with chronic lung damage, history of viral CNS infections, and very low IgA or IgM levels. For example, two of three patients with enteroviral meningoencephalitis recovered from infections with higher dose of IVIG achieving trough levels between 3,100 and 6,300 mg/dL (8). High dose IgRT, however, had no beneficial effect in severe Norovirus infection in patients with CVID (99).

A recent systematic review and meta-analysis showed that weekly SCIG therapy resulted in higher IgG trough levels, and a linear decrease in incidence of infections with every 100 mg/dl increase in IgG trough levels. This was not observed in patients who received IVIG (164). Another recent study found that IVIG rather than SCIG therapy was associated with higher risk of bronchiectasis (165). This observation require confirmation by larger, controlled studies.

## Other Therapies to Prevent Infections

Prophylactic antibiotics have been used to prevent infections in PADs (166). However, their negative effect on airway and gastrointestinal microbiota continues to be a major concern (159).

Administration of conjugated pneumococcal vaccine may achieve protective antibody levels and prevent pneumococcal infections in SIAD and IgSCD.

Although hematopoietic stem cell transplantation is not recommended in PADs, mostly due to the risks of GVHD, associated mortality, or no benefit, reduced intensity conditioning showed promising results in XLA (167, 168).

## Author Contributions

YY prepared the first draft of the manuscript. YY and SG contributed to editing and reviewed and authorized the final version. All authors contributed to the article and approved the submitted version.

## Conflict of Interest

The authors declare that the research was conducted in the absence of any commercial or financial relationships that could be construed as a potential conflict of interest.
